# Prognostic value of pretreatment serum albumin−globulin ratio in urothelial carcinoma: A systematic review and meta-analysis

**DOI:** 10.3389/fonc.2022.992118

**Published:** 2022-08-16

**Authors:** Zhongyou Xia, Xueqin Fu, Jinze Li, Ji Wu, Chao Niu, Yulai Xu, Hao Wang, Xinzhu Yuan, Lingtong Tang

**Affiliations:** ^1^ Department of Urology, Nanchong Central Hospital, The Second Clinical College, North Sichuan Medical College (University), Nanchong, China; ^2^ Department of Breast Surgery, Guizhou Provincial People’s Hospital, Guiyang, China; ^3^ Department of Urology, Institute of Urology, West China Hospital, Sichuan University, Chengdu, China; ^4^ Blood Purification Center of Department of Nephrology, Nanchong Central Hospital, The Second Clinical College, North Sichuan Medical College (University), Nanchong, China; ^5^ Department of Clinical Laboratory, The People’s Hospital of Gao County, Yibin, China

**Keywords:** bladder cancer, upper tract urothelial carcinoma, urothelial cancer, albumin−globulin ratio, prognosis

## Abstract

**Objective:**

To evaluate whether pretreatment albumin−globulin ratio (AGR) can be used as a biomarker for predicting the prognosis of patients with urothelial carcinoma (UC).

**Methods:**

We systematically searched PubMed, Web of Science, China National Knowledge Infrastructure (CNKI), Google Scholar and Cochrane Library; the search time was up to May 2022. Stata 16.0 was used for data processing and statistical analysis.

**Results:**

We identified 12 studies with 5,727 patients from 317 unique citations during the meta-analysis. Our results suggested that a low AGR before treatment was significantly associated with poor overall survival (OS) [hazard ratio (HR) = 1.99, 95% confidence interval (CI) = 1.45-2.75, P < 0.001], cancer-specific survival (CSS) [HR=2.01, 95% CI = 1.50-2.69, P < 0.001] and recurrence-free survival (RFS) [HR=1.39, 95% CI = 1.12-1.72, P = 0.002]. Furthermore, we defined different subgroups according to ethnicity, cancer type, cut-off value, sample size and stage. Similar prognostic outcomes for OS and CSS were observed in most subgroups. However, for subgroup of stage, the low pretreatment AGR only predicted the poor survival of patients with non-metastatic UC.

**Conclusion:**

Our meta-analysis revealed that the AGR before treatment could be used as a predictive biomarker to indicate the prognosis of UC patients during clinical practice, especially in patients with non-metastatic UC.

## Introduction

Urothelial carcinoma (UC) mainly originates in the bladder, renal pelvis, ureters, and urethra and is the fourth most frequently diagnosed cancer worldwide (1). It has been reported that 90% of UCs occur in the bladder, with bladder cancer (BC) being the most common UC (2). Moreover, according to a World Health Organization (WHO) report, there will be 573,278 new cases and 212,536 deaths BC-related deaths worldwide in 2020. Conversely, upper tract urothelial carcinoma (UTUC) is rare and only accounts for only 5%-10% of all UC patients with a poor prognosis (3). BC can be divided into three subtypes: non-muscle-invasive bladder cancer (NMIBC), muscle-invasive bladder cancer (MIBC), and metastatic BC. Nearly 75% of bladder cancers initially present with NMIBC. Intravesical chemotherapy or intravesical immunotherapy after transurethral resection is the standard treatment for NMIBC (4, 5). In contract, radical cystectomy (RC) with extended pelvic lymphadenectomy is the mainstay of treatment for patients with MIBC (6). However, BC, which has a severe recurrence and progression rate, and an estimated 5-year overall survival (OS) of only 10% to 40%, cannot be entirely overcome by definitive treatment (7). UTUC is characterized by a high degree of malignancy. Radical nephroureterectomy (RNU) with bladder cuffing is the standard treatment for high risk nonmetastatic UTUC patients, but 20%-30% of patients have distant metastasis and a poor survival rate of 5 years after surgery (8, 9). Hence, a valuable prognostic indicator should be identified to predict the survival and recurrence in patients with UC.

In addition to traditional prognostic factors such as tumor TNM stage, grade, lymphovascular invasion (LVI), and node classification, numerous clinical trials have found that smoking history, sex, symptoms and other clinical prognostic factors have also been used to evaluate the prognosis of UC patients (10–12). However, these clinical and pathological factors remain limited in improving outcome predictions for UC patients. Recently, several meta−analysis have identified that preoperative laboratory hematological biomarkers, including C-reactive protein (CRP), hemoglobin, lymphocyte-monocyte ratio (LMR), neutrophil-to-lymphocyte ratio (NLR), platelet-lymphocyte ratio (PLR), white blood cell count, and De Ritis ratio, may have prognostic value in patients with UC (13, 14).

Albumin and globulin are the main proteins in the serum and closely related to nutritional status and systemic inflammation in cancer patients (15). The albumin-to-globulin ratio (AGR) is a ratio that combines the two indexes (albumin and globulin). Recent studies have revealed that AGR is an independent prognostic factor for several cancers, such as multiple myeloma (16), non-small cell lung cancer (17), and colorectal cancer (18). Furthermore, low AGR is associated with worse survival. However, the use of AGR to evaluate the clinical prognosis of patients with UC remains controversial. Although the published literature shows that UC patients with low AGR have a poorer prognosis, Pradere et al. (19) discovered that pretreatment AGR is not associated with OS or recurrence-free survival (RFS). Accordingly, our study aimed to reveal the prognostic value of pretreatment AGR and to guide our clinical practice.

## Materials and methods

Our study was performed according to the Preferred Reporting Items for Systematic Reviews and Meta-Analyses (PRISMA) guideline (20).

### Literature search

PubMed, Web of Science, China National Knowledge Infrastructure (CNKI), Google Scholar, and Cochrane Library were used to search related published articles that assessed the prognostic value of AGR for UC patients. The time range of the literature search was up to May of 2022. All searches were limited to human studies and no language restrictions were applied.

The search terms were as follows: “urothelial carcinoma”, “urothelial cancer”, “bladder cancer”, “upper tract urothelial carcinoma”, “upper tract urothelial cancer”, “UTUC”, “prognosis”, “prognostic factors”, “survival”, “serum albumin”, “serum globulins”, “albumin to globulin ratio”, “albumin/globulin ratio” and “AGR” as Mesh term or keywords. The above search fields were randomly combined to achieve a complete search. Furthermore, two authors independently performed the searches, and a third author resolved any discrepancies.

### Inclusion and exclusion criteria

The included researches should meet the following criteria: (1) patients pathologically diagnosed with UC (including bladder cancer or upper tract urothelial carcinoma); (2) Provision of an exact AGR cut-off value before receiving treatment; (3) hazard ratio (HR) and related 95% confidence intervals (CIs) were provided; (4) corresponding survival outcomes, such as overall survival (OS), cancer-specific survival (CSS), recurrence-free survival (RFS), tumor-specific survival (TSS), progression-free survival (PFS), metastasis-free survival (MFS), had been reported; (5) studies with randomized controlled trials (RCTs), case-control studies, or cohort studies.

Studies were excluded based on the following criteria: review articles, repetitive reviews, letters, case reports, studies unrelated to the subject matter, duplicated studies based on the same patients, and studies with no detailed data.

### Data extraction and quality assessment

Two researchers separately extracted the relevant data from the included literatures and resolved the disagreements through negotiation or by a senior author. The outcomes of interest for each included study were as follows: first author’s name, country, study design, sample size, intervention, age, cancer type, cut-off value for AGR, follow-up time, and outcome indicators. When univariate and multivariate analyses were conducted in a study, we extracted the multivariate analysis data for follow-up analysis. Based on preliminary search results, the Newcastle-Ottawa Scale (NOS) was used to evaluate the quality of the included studies (21). According to the evaluation of the three question areas of selection, comparability, and exposure in the scale, a score of more than six stars was considered to indicate high-quality research.

### Statistical analysis

In this study, Stata 16 (StataCorp LP, University City, Texas, USA) was used for the statistical analysis. Multivariate HR and corresponding 95% CI were extracted from each study, and data were synthesized to assess the prognostic value of AGR on UC patients survival. The heterogeneity between the included studies was verified using Cochranes Q test and I^2^ test. Regarding the heterogeneity test results, the random-effects model was used when heterogeneity was present (I^2^ ≥ 50% or p < 0.1). Otherwise, fixed-effects models were used for the analyses. I^2^ > 50% indicated significant heterogeneity between studies. We performed subgroup analyses based on ethnicity, cancer type, cutoff value, sample size and stage to evaluate the heterogeneity. Sensitivity analysis, involving the removal of each individual study, was also used to assess the robustness of our survival outcomes. Additionally, Begg’s test was performed to explore potential publication bias. value of P less than 0.05 was considered statistically significant.

## Results

### Description of studies and quality assessment

**Figure 1:**
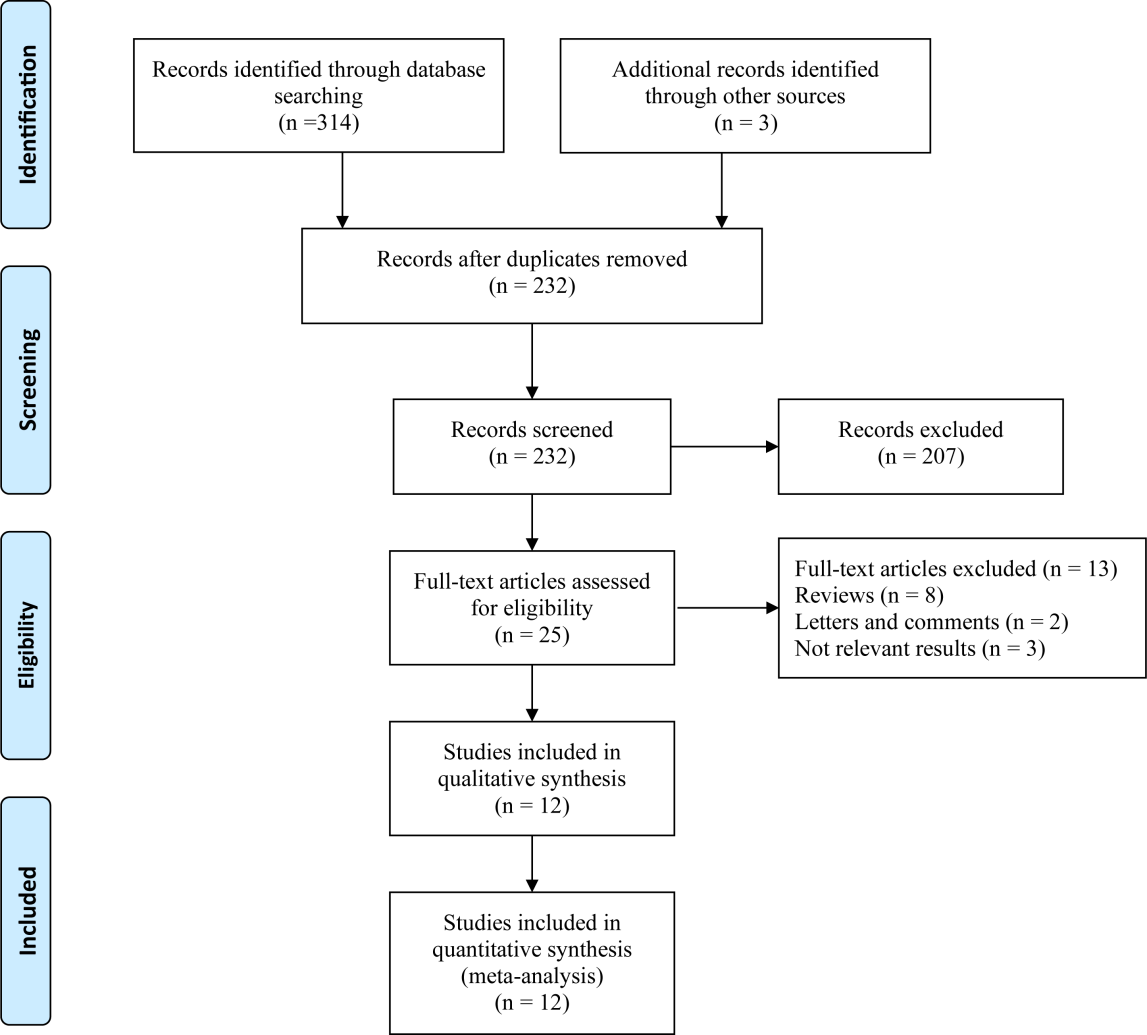
Flow diagram of studies selection process.

In total, 317 articles were retrieved from the databases and manually searched. After removing the relevant repeated studies, 232 studies remained. Subsequently, 207 articles were excluded from the analysis of research topics and abstracts. Full-text analysis was carried out for the remaining 25 eligible studies; relevant data could not be extracted from eight reviews, two letters and comments, and three articles. Finally, 12 articles with 5,727 patients were included in this study for further analysis (**Figure 1**).

The baseline data are presented in **Table 1**. Eleven retrospective articles and one propensity-matched scoring study were included. All the studies have focused on bladder urothelial cancer and upper tract urothelial carcinoma. The included literatures were single-center or multicenter studies, which were published between 2015 and 2021. The cutoff values for the AGR ranged from 0.95 to 1.55. The mean follow-up time ranged from 7.5 to 78 months. Additionally, all included studies with a NOS score of 6 or higher were regarded as high-quality studies (**Table 1**).

**Table 1:** Baseline characteristics of include studies and methodological assessment.

Authors (year)	Region	Study Design	Sample Size	Intervention	Age^a^	Cancer Type	Stage	Cutoff Value	Follow-Up Time^c^ (months)	Outcome Indicators	Quality Score
Zhang 2015 (22)	China	Retrospective	187	RNU	70 (61–74)	UTUC	N	1.45	Median 78 (32-92)	OS, CSS	8
Liu 2016 (23)	China	Retrospective	296	RC	61.71 ± 11.08	BC	N	1.6	Median 72.0 (49.75-115.50)	RFS, CSS	7
Liu 2017 (24)	China	PSM	104	RC	NA	BC	N	1.55	Median 38 (1-90)	OS, PFS, TSS	7
Fukushima 2018 (25)	Japan	Retrospective	105	RNU	74 (49-89)	UTUC	N	1.24	Median 46 (22-83)	OS, DFS	6
Otsuka 2018 (26)	Japan	Retrospective	124	RNU	69 (64–75)	UTUC	N	1.4	Median 55 (28-76)	OS, RFS, CSS	7
Xu 2018 (27)	China	Retrospective	620	RNU	NA	UTUC	N	1.45	Median 50 (28-78)	RFS, CSS, OS	8
Omura 2020 (28)	Japan	Retrospective	179	RNU	75 (66-79)	UTUC	N	1.25	Median 34 (17-63)	OS, CSS	7
Oh 2021 (29)	Korea	Retrospective	176	RC	68.05 ± 8.96	BC	N	1.32	Median 32.4 (0.2-95.3)	CSS, MFS	8
Quhal2021 (30)	multicenter	Retrospective	1096	TURBT	67 (58-74)	BC	N	1.41	Median 63.7 (25.3-111)	PFS, RFS	8
Miura 2021 (31)	multicenter	Retrospective	2492	RNU	69 (27-97)	UTUC	N	1.4	Median 38	RFS, CSS, OS	7
Pradere 2021 (19)	multicenter	Retrospective	172	NAC+RNU	68 (63-73)	UTUC	N	1.42	Median 26 (11-56)	OS, RFS	8
Taguchi 2021 (32)	multicenter	Retrospective	176	pembrolizumab	71 (66-76)	Mix^b^	N+M	0.95	Median 7.5 (4-14)	OS, CSS, PFS	7

a Age, Mean ± SD/Mean (Range).

b Mix, Bladder Cancer, Upper tract urothelial carcinoma.

c Follow-up Time, Median (Range)/Median; PSM, propensity score-matched; RNU, Radical nephroureterectomy; BC, Bladder Cancer; UTUC, Upper tract urothelial carcinoma; N, non-metastatic; N+M, non-metastatic+metastatic; TURBT, Transurethral resection of bladder tumor; NAC, neoadjuvant chemotherapy; OS, overall survival; CSS, cancer-specific survival; RFS, recurrence-free survival; TSS, tumor-specific survival; PFS, progression-free survival; MFS, metastasis-free survival; NA, age data was not available.

### Association of AGR with OS

**Figure 2:**
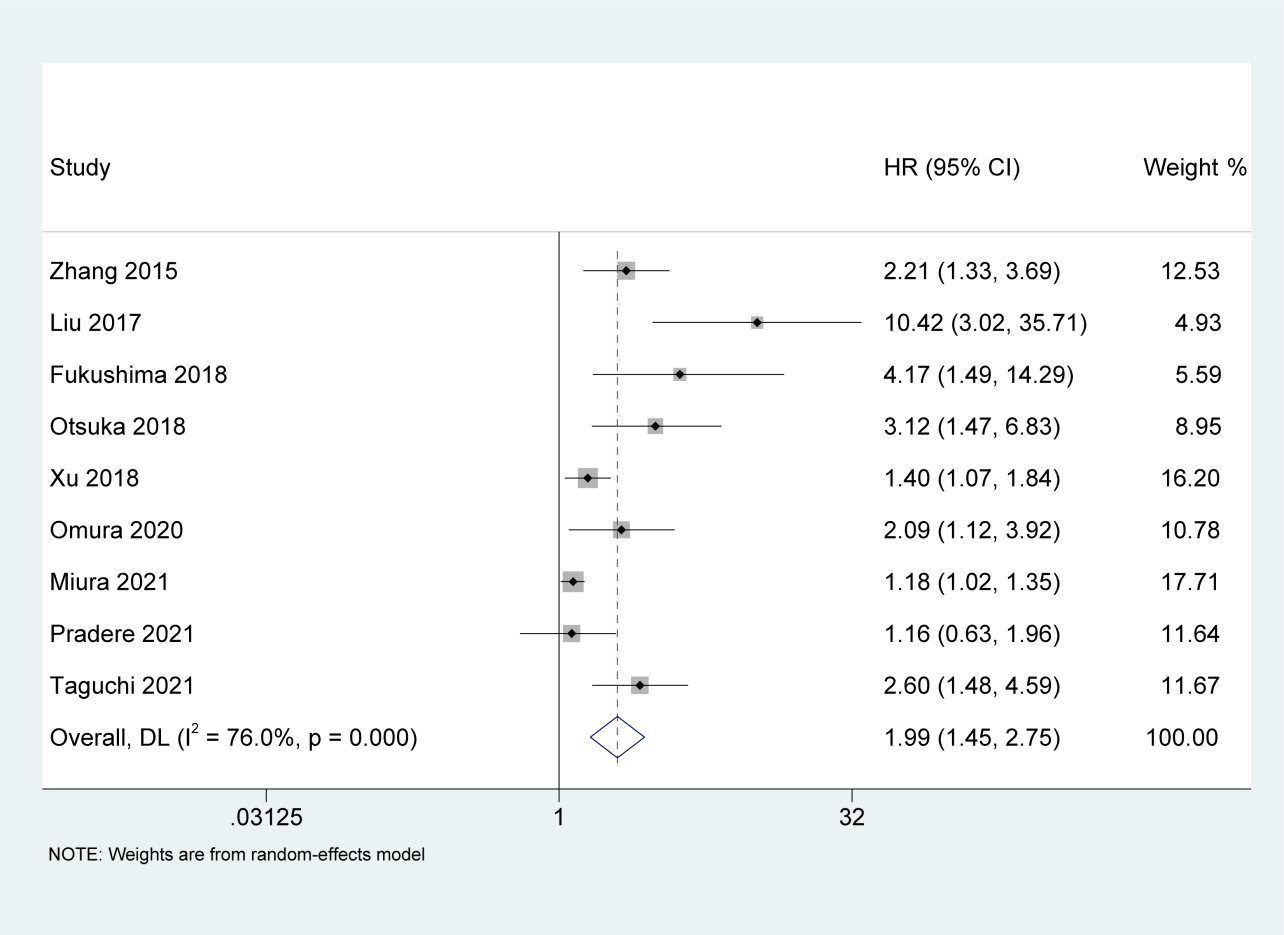
Forest plot and meta-analysis of the association between overall survival and albumin to globulin ratio.

Nine studies (19, 22, 24–28, 31, 32) reported an association between pretreatment AGR and OS in UC patients. Owing to the heterogeneity test outcome (I^2^ = 76%, p < 0.001), we used a random-effects model. Our meta-analysis revealed that compared with the high AGR group, the low AGR group had inferior OS, and the difference between the two groups was statistically significant [HR = 1.99, 95% CI (1.45-2.75), p < 0.001, **Figure 2**].

### Association of AGR with CSS

**Figure 3:**
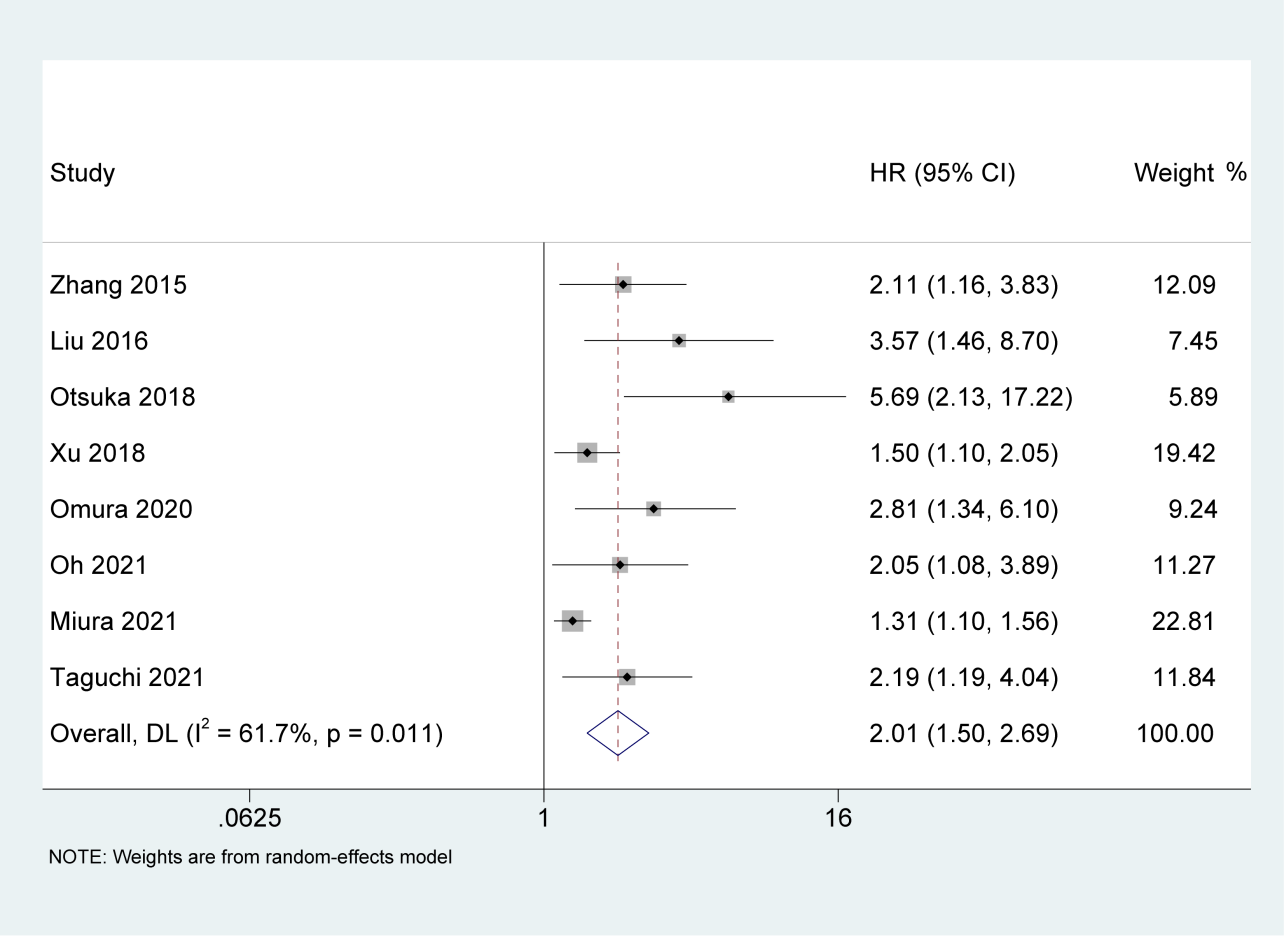
Forest plot and meta-analysis of the association between cancer-specific survival and albumin to globulin ratio.

Study reports from eight studies (22, 23, 26–29, 31, 32), with 4,250 patients enrolled, indicated the prognostic value of AGR in patients with UC on CSS. The pooled results indicated that the lower pretreatment AGR was correlated with poorer CSS [HR = 2.01, 95% CI (1.50-2.69), P < 0.001, **Figure 3**], with a high heterogeneity (I^2^ = 61.7%, p = 0.011).

### Association of AGR with RFS

**Figure 4:**
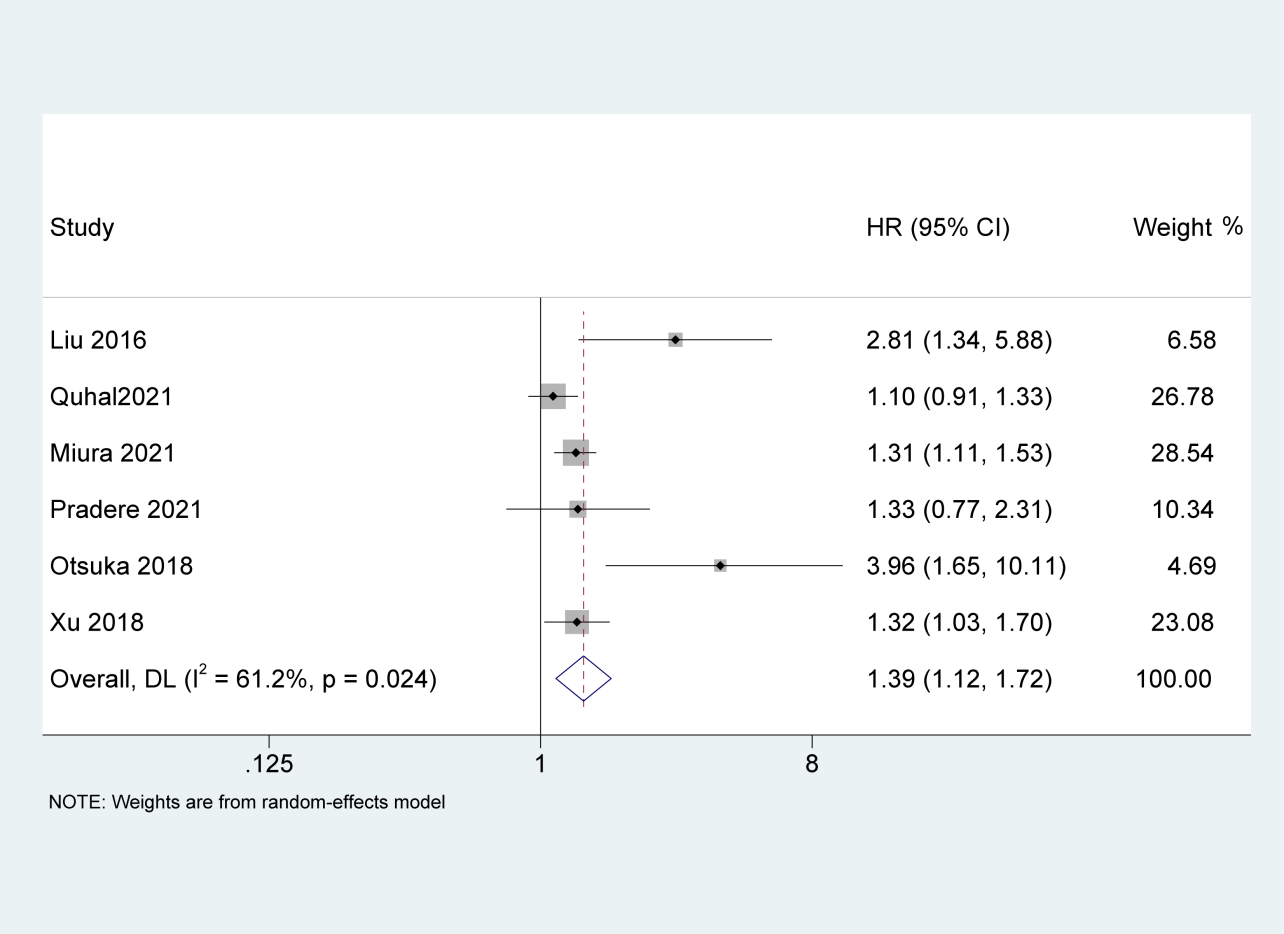
Forest plot and meta-analysis of the association between recurrence-free survival and albumin to globulin ratio.

Six studies (19, 23, 26, 27, 30, 31) with 4,628 patients recorded the impact of pretreatment AGR on RFS. Because of the high heterogeneity between the studies (I^2^ = 61.2%, p = 0.024), a random-effects model was used. The results of the meta-analysis demonstrated that pretreatment AGR was an independent risk factor for poor RFS in patients with UC [HR = 1.39, 95% CI (1.12-1.72), P = 0.002, **Figure 4**].

### Subgroup analysis

We conducted a subgroup analysis for OS and CSS with limited studies, and stratified by ethnicity, cancer type, cutoff value, and sample size (**Table 2**). Stratified according to the ethnicity, in the Chinese population, the low pretreatment AGR was positively correlated with poor OS [HR = 2.51, 95% CI (1.17, 5.38), P = 0.018] and CSS [HR=1.95, 95%CI (1.25, 3.02), P=0.003]. Similarly, in other regional subgroups, we found that a low AGR was an independent risk factor for OS [HR = 1.81, 95% CI (1.25, 2.93), P = 0.003] and CSS [HR = 2.17, 95% CI (1.36, 3.47), P=0.001]. However, low AGR was not related to RFS in the Chinese population. Subgroup analyses stratified by cancer type, our results showed that the low AGR predicted a worse OS [HR = 1.63, 95% CI (1.24, 2.13), p < 0.001] and CSS [HR = 1.86, 95% CI (1.31, 2.64), P = 0.001] in the UTUC group. The same results were observed in BC group [OS: HR = 10.42, 95% CI (3.02, 35.71), p < 0.001; CSS: HR=2.48, 95%CI (1.47, 4.16), P=0.001] and mix group [OS: HR = 2.60, 95% CI (1.48, 4.59), P = 0.001; CSS: HR = 2.19, 95% CI (1.19, 4.04), P = 0.001] with limited articles. For subgroup of cut-off value, the meta-analysis demonstrated that in the cut-off value <1.41 [OS: HR = 2.19, 95% CI (1.28, 3.73), P = 0.004; CSS: HR = 2.17, 95% CI (1.36, 3.47), P = 0.001] and cut-off value ≥1.41 [OS: HR = 1.89, 95% CI (1.13, 3.16), P = 0.017; CSS: HR = 1.95, 95% CI (1.25, 3.02), P = 0.003], all the above results indicated that the low pretreatment AGR was related to poor OS and CSS. Stratified by sample size, lower OS and CSS were more likely to be related to low AGR, either in the sample size ≥179 group [OS: HR = 1.50, 95% CI (1.14, 1.97), P = 0.005; CSS: HR = 1.75, 95% CI (1.29, 2.38), p < 0.001] or sample size <179 [OS: HR = 2.90, 95% CI (1.46, 5.73), P = 0.001; CSS: HR = 2.56, 95% CI (1.54, 4.26), p < 0.001]. In addition, stratified by stage, due to limited data, we only observed that low AGR was associated with a poor OS [HR = 1.86, 95% CI (1.35,2.57), p < 0.001] and CSS [HR = 1.81, 95% CI (1.46, 2.75), p < 0.001] in patients with non-metastatic UC.

**Table 2:** Subgroup analysis of OS and CSS based on different influencing factors.

Subgroups	Variable	No. of studies	Effect model	HR (95%CI)	P	Heterogeneity	
						I^2^ (%)	p
**OS**	All	9	Random	1.99 (1.45, 2.75)	< 0.001	76.0%	< 0.001
Ethnicity	China	3	Random	2.51 (1.17, 5.38)	0.018	82.1%	0.004
	others	6	Random	1.81 (1.25, 2.93)	0.003	73.8%	0.002
Cancer type	BC	1	Fix	10.42 (3.02, 35.71)	< 0.001	NA	NA
	UTUC	7	Random	1.63 (1.24, 2.13)	< 0.001	66.1%	0.007
	Mix	1	Fix	2.60 (1.48, 4.59)	0.001	NA	NA
Cutoff value	≥1.41	4	Random	1.96 (1.13, 3.40)	0.017	76.0%	0.006
	<1.41	5	Random	2.19 (1.28, 3.73)	0.004	78.8%	0.001
Sample size	≥179	4	Random	1.50 (1.14, 1.97)	0.004	64.7%	0.037
	<179	5	Random	2.91 (1.54, 5.50)	0.001	69.6%	0.010
Stage	N	8	Random	1.86 (1.35, 2.57)	< 0.001	75.5%	< 0.001
	N+M	1	Fix	2.6 (1.48, 4.58)	0.001	NA	NA
**CSS**	All	8	Random	2.01 (1.50, 2.69)	< 0.001	61.7%	0.011
Ethnicity	China	3	Random	1.95 (1.25, 3.02)	0.003	46.9%	0.152
	others	5	Random	2.17 (1.36, 3.47)	0.001	70.5%	0.009
Cancer type	BC	2	Random	2.48 (1.47, 4.16)	0.001	0.0%	0.321
	UTUC	5	Random	1.86 (1.31, 2.64)	0.001	67.6%	0.015
	Mix	1	Fix	2.19 (1.19, 4.04)	0.012	NA	NA
Cutoff value	≥1.41	3	Random	1.95 (1.25, 3.02)	0.003	46.9%	0.152
	<1.41	5	Random	2.17 (1.36, 3.47)	0.001	70.5%	0.009
Sample size	≥179	5	Random	1.75 (1.29, 2.38)	< 0.001	58.8%	0.046
	<179	3	Random	2.56 (1.54, 4.26)	< 0.001	31.5%	0.232
Stage	N	7	Random	2.0 (1.46, 2.75)	< 0.001	64.6%	0.009
	N+M	1	Fix	2.19(1.19, 4.04)	0.012	NA	NA

BC, bladder cancer; UTUC, upper tract urothelial carcinoma; Mixed, bladder cancer and upper tract urothelial carcinoma; NA, only one study was included in the subgroup, and the heterogeneity test could not be performed; N, non-metastatic, N+M, mon-metastatic+metastatic.

### Sensitivity analysis

Because of the high heterogeneity of some parameters, we performed a sensitivity analysis for the pooled HRs of OS and CSS. No significant change in the pooled HR was observed according to the leave-one-out test. Hence, we believe that our results are reliable (**Figure S1**).

### Publication bias

Begg’s test was performed to assess the publication bias in this study. Visual examination of the funnel plot and statistical analysis revealed potential publication bias was exist for OS (p = 0.048) and CSS (p = 0.004). Subsequently, a test of trim and fill in STATA using the “metatrim” command was adopted to further investigate the publication bias; furthermore, there were four potentially missing studies according to the filled funnel plot. However, our pooled results basically did not change after adding four unpublished studies (**Figure S2**).

## Discussion

UC is a highly invasive tumor with a high mortality rate. Even in cases where patients received radical surgery, 20%-30% of them still had a recurrence and distant metastasis that seriously affected their quality of life (33–35). As common predictive indicators for UC, including tumor stage, grade and lymph node status, it is difficult to accurately predict the prognosis of patients before treatment alone (13). In recent years, researchers have developed nomograms to predict the survival outcomes in a variety of urinary cancer patients. Zhang et al. (36) developed a nomogram including the clinicopathological features, AGR, C-reactive protein/albumin ratio (CAR), and neutrophil-lymphocyte ratio (NLR) to predict OS and PFS after radical surgery for bladder cancer. Chen et al. used the data of T stage, AGR, NLR, and monocyte to lymphocyte ratio (MLR) to construct a nomogram and use it to evaluate the survival outcome of clear cell renal cell carcinoma (ccRCC) patients (37). Although the nomogram provides an accurate prediction, we need to incorporate multiple factors that will increase the financial burden of patients. Additionally, some pathological indicators need to be obtained by surgery, which will bring trauma to the patients. Therefore, identifying a noninvasive, inexpensive and easily accessible prognostic biomarker is of great significance in guiding the individual treatment of patients with UC.

Recently, studies have shown that AGR can be used as an economic and practical marker to evaluate the therapeutic effect and prognosis of different cancers (15–17, 38). Similarly, for UC patients, most studies identified that AGR before treatment was an independent prognostic factor in multivariate analysis (22–29, 31, 32, 39). However, the prognostic value of AGR remains controversial in some clinical studies (19, 30). Although the previous two meta-analyses reported that low AGR had poor survival (13, 40), the reliability of their research conclusions was insufficient because only two studies were included. Thus, it is necessary to use the published literatures to further clarify the value of pretreatment AGR in the clinical diagnosis and prognosis of patients with UC.

In the current study, we performed a meta-analysis on 12 articles involving 5,727 patients to explore the prognostic effect of AGR in UC patients. According to the pooled analysis results, this conclusion was in line with most of the included studies, which suggested that pretreatment AGR was an independent predictor of survival outcomes. With a decrease of AGR, patients with UC had worse OS, CSS and RFS outcomes. Subgroup analyses of OS and CSS by race, cancer type, cutoff, or sample size also showed that low AGR was significantly associated with poorer OS and CSS in all UC patients. However, for tumor stage, limited data was used to demonstrated that low AGR was an independent prognostic marker for patients with non-metastatic UC.

It is worth noting that sensitivity analysis confirmed that the pooled results were stable. However, from the Cochrane’s Q test and I^2^ test, we found that moderate to extreme inter-study heterogeneity for survival outcomes among the included studies. Hence, a random effects model was used to minimize the impact of heterogeneity on the overall effects. This discrepancy might be because the population of participants used for the study was limited to Asia. Moreover, there were different treatment regimens and subsequent treatment approaches.

Albumin and globulins are the two most abundant proteins in human blood plasma and can be easily and cost-effectively measured. As the major serum protein, albumin plays an vital role in carrying out antioxidant activities, maintaining colloidal osmotic pressure, binding and transporting hormones, cations and fatty acids, as well as in maintaining capillary membrane stability (41, 42). Similarly, the albumin level either reflects the body nutritional status or represents the systemic inflammation (43). Overall, the inflammatory response to cancer is strongly linked to its prognosis and can be used to predict the clinical course (44, 45). Inflammation plays a pivotal step in cancer occurrence and development. In an inflammatory environment, tumor cells release various inflammatory mediators, such as tumor necrosis factor (TNF), interleukin-1 and interleukin-6, and increase vascular permeability by damaging vascular endothelial cells. In contrast, malnutrition and inflammatory cytokines can inhibit the production of albumin, resulting in low serum albumin concentration, which could influence cell proliferation and weaken human immune defense mechanisms (33, 43). Globulin contains several immune-related proteins, such as C-reactive protein, complement components, fibrinogen and serum amyloid A, which are involved in regulating immunity and inflammation (27, 32). Chronic inflammation can affect not only the tumor growth but also angiogenesis and cancer migration (46). Previous studies have demonstrated that pre-treatment albumin and globulin are two potentially valuable elements related to the prognosis and can define the risk stratification of cancer patients (33, 47, 48).

Unfortunately, when albumin and globulin are used alone as an evaluation indices, unstable results may occur and easily interfered with external confounding factors. Consequently, we speculate that AGR combines two independent prognostic parameters, which have a higher predictive value than serum albumin or globulin levels alone. Several clinical trials have reported that AGR could be as a potential prognostic biomarker and that the decrease in AGR was correlates significantly with tumor stage and grade in different human cancers, including urinary system cancers (16, 31, 49, 50). Previous meta-analyses have also clarified the prognostic value of the AGR in various solid tumors. A meta-analysis conducted by Lv et al. (40) discovered that decreased AGR resulted in worse OS and PFS, and increased cancer recurrence or progression in many malignancies. In colorectal cancer, Ma et al. (51) provided evidence that low pretreatment AGR was related poor OS (HR=2.07, *P* < 0.01) and DFS/PFS (HR=2.10, *P* = 0.01), and advanced clinicopathological features, including age, tumor size, node metastasis stage, tumor depth. Additionally, many multicenter studies successively explored the correlation between the AGR and UC in recent years. A multicenter research team performed a retrospective study involving 2492 patients with non-metastatic UTUC receiving radical nephroureterectomy (RNU) and demonstrated that lower preoperative AGR is associated with locally advanced disease and worse clinical outcomes (31). In Pradere’s study of patients with UTUC undergoing neoadjuvant platin-based chemotherapy and RNU, the patients with a low pretreatment AGR had a markedly shorter OS and RFS than those with a high AGR group (19). In 2021, the finding of Taguchi and his colleagues proved that the AGR before treatment could assist in predicting the survival of UC patients treated with pembrolizumab (32). According to the current analysis, our results further revealed that low pretreatment AGR provides an accurate prognostic efficiency for OS, CSS, and RFS in UC patients. Therefore, we recommend that AGR could act as an efficient prognostic indicator for UC.

While our study contributes important evidence around the prognostic value of AGR, it has some limitations. Firstly, most of the included studies had a relatively small sample size. Secondly, since all studies were retrospective in design and most of the population came from Asia, the evidence obtained was limited. Thirdly, different treatment strategies may introduce bias in the results. Fourthly, the cutoff values of pretreatment AGR was inconsistent in different studies, which also led to bias in our results. Lastly, since other clinically relevant pathological data, such as tumor stage and grade were not available, further subgroup analysis could not be performed.

## Conclusions

According to our analysis, this meta-analysis reveals that the pretreatment AGR is an independent prognostic indicator for patients with UC, especially for non-metastatic UC patients. Low AGR is related to worse OS, CSS and RFS. However, future studies with larger sample sizes and randomized controlled trials are needed to confirm this conclusion.

## Data availability statement

The original contributions presented in the study are included in the article/**Supplementary Material**. Further inquiries can be directed to the corresponding authors.

## Author contributions

They conceived and designed the experiments: LT and XY. Analysed the data: ZX, XF, JL, and JW. Contributed reagents/materials/analysis: JL, CN, YX, and HW. Wrote the manuscript: ZX and XF. All authors contributed to the article and approved the submitted version.

## Acknowledgments

The authors thank Editage (www.editage.cn) for English language editing.

## Conflict of interest

The authors declare that the research was conducted in the absence of any commercial or financial relationships that could be construed as a potential conflict of interest.

## Publisher’s note

All claims expressed in this article are solely those of the authors and do not necessarily represent those of their affiliated organizations, or those of the publisher, the editors and the reviewers. Any product that may be evaluated in this article, or claim that may be made by its manufacturer, is not guaranteed or endorsed by the publisher.

## Supplementary material

Supplementary Figure 1 Forest plot for sensitivity analysis. **(A)** overall survival and **(B)** cancer-specific survival.

Supplementary Figure 2 Begg’s test for **(A)** overall survival and **(B)** cancer-specific survival; Trim and fill method for **(C)** overall survival and **(D)** cancer-specific survival.
