# Supplementary figures and images for: Prognostic value of pretreatment serum albumin−globulin ratio in urothelial carcinoma: A systematic review and meta-analysis
Source: Front Oncol. 2022 Aug 16;12:992118. doi: 10.3389/fonc.2022.992118 (PMC9424645; doi:10.3389/fonc.2022.992118)

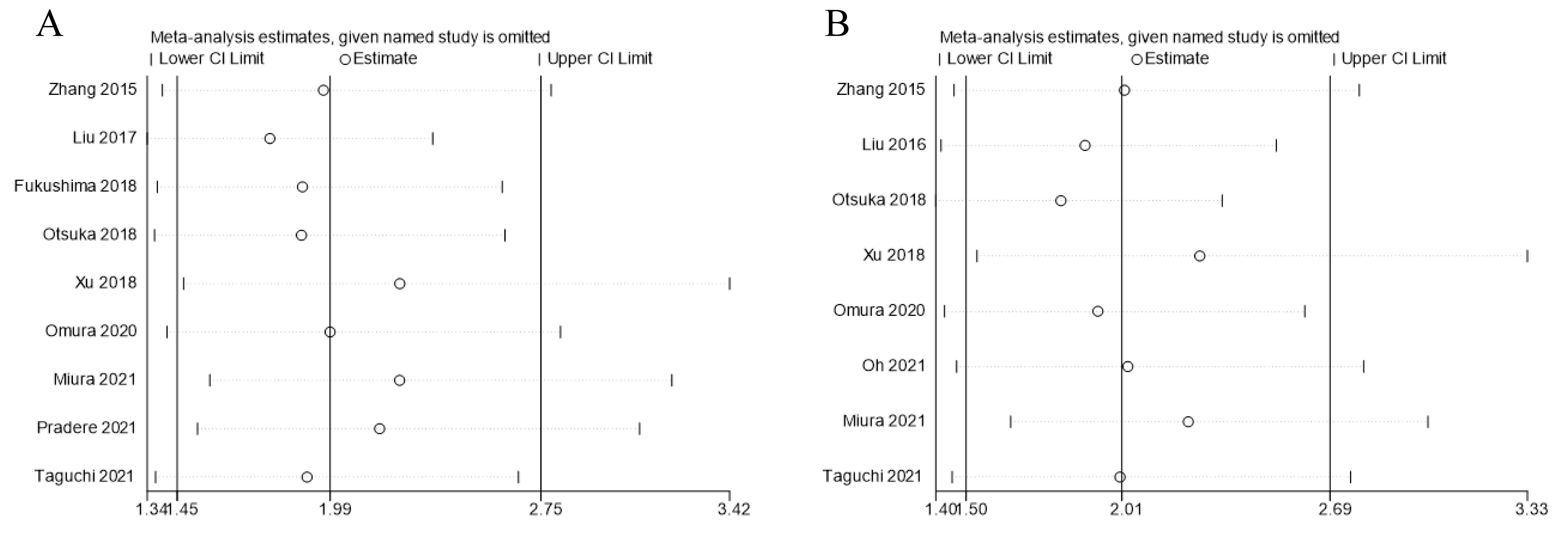

Supplement: Supplementary Figure 1 — Forest plot for sensitivity analysis. (A) overall survival and (B) cancer-specific survival. [file Image_1.tif]

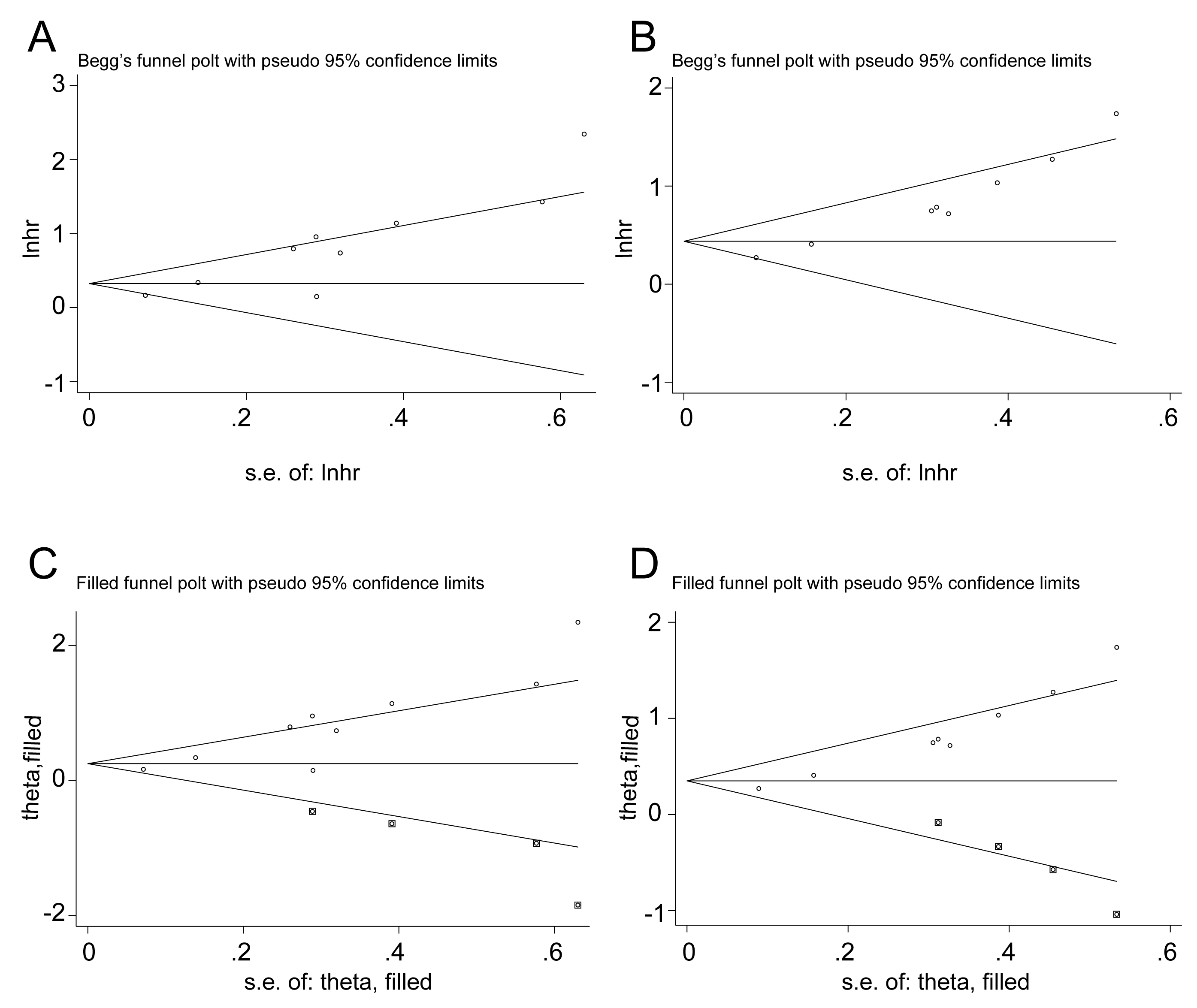

Supplement: Supplementary Figure 2 — Begg’s test for (A) overall survival and (B) cancer-specific survival; Trim and fill method for (C) overall survival and (D) cancer-specific survival. [file Image_2.tif]
